# Prevalence and Risk Factors Comparison of Anterior and Posterior Intracranial Arterial Stenosis

**DOI:** 10.1155/2022/7710374

**Published:** 2022-01-10

**Authors:** Yan Zhao, Beibei Liu, Chunxiu Wang, Shaochen Guan, Chunxiao Liu, Yanlei Zhang, Chengbei Hou, Xiaowei Song, Zhongying Zhang, Xiaoguang Wu, Huihui Li, Xiang Gu, Shimin Hu, Jian Wu, Xianghua Fang

**Affiliations:** ^1^Department of Geriatrics, Xuanwu Hospital of Capital Medical University, Xicheng District, Beijing 100053, China; ^2^Department of Vascular Ultrasound, Xuanwu Hospital of Capital Medical University, Xicheng District 100053, Beijing, China; ^3^Evidence Based Medicine Center, Xuanwu Hospital of Capital Medical University, 45 Changchun Street, Xicheng District, Beijing 100053, China; ^4^Department of Neurology, Beijing Tsinghua Changgung Hospital, School of Clinical Medicine, Tsinghua University, Changping District, Beijing 102218, China; ^5^Department of Geriatrics, Beijing Friendship Hospital, Capital Medical University, Beijing 100050, China; ^6^Department of Neurology, Xuanwu Hospital of Capital Medical University, 45 Changchun Street, Xicheng District, Beijing 100053, China; ^7^School of Clinical Medicine, Tsinghua University, 30 Shuangqing Road, Haidian District, Beijing 100084, China

## Abstract

The prevalence and risk factors of intracranial atherosclerotic stenosis (ICAS) located in the anterior circulation (AC) and posterior circulation (PC) has been scarcely noted in the general population. We aimed to determine ICAS prevalence and risk factor profile of AC and PC in a representative population. Data were from the China Hypertension Survey of Beijing. In total, 4800 people aged 35 years or older were enrolled in this subsurvey for ICAS, and 3954 participants were eligible for analysis. ICAS was assessed by transcranial Doppler. The prevalence of ICAS in AC was much greater than that in PC (11.9% vs. 4.2%), and subjects with ICAS in PC were 3.9 years older than those with ICAS in AC. Multivariable logistics regression showed that the odds of hypertension and diabetes increased by 79% (OR: 1.79, 95% CI: 1.40–2.27) and 35% (OR: 1.35, 95% CI: 1.04–1.75) in those with AC vascular lesions and by 3.35 times (OR: 3.35, 95% CI: 2.49–4.50) and 71% (OR: 1.71, 95% CI: 1.19–2.46) in those with PC vascular lesions compared with those without vascular lesions. Most modifiable vascular risk factors for ICAS appeared to exert similar magnitudes of risk for PC to AC lesions.

## 1. Introduction

The incidence of stroke in China has increased over the last decades [1], and the rise has mainly been attributed to ischemic stroke, with an annual growth rate of 8.7% [2]. Intracranial atherosclerotic stenosis (ICAS) is a predominant cause for ischemic stroke, particularly for the Asian population. The percentage of ICAS ranged from 37% to 46.6% in ischemic stroke patients from China [3–7] and 30% in patients from Korea [3–7], which is higher than that of western countries of ICAS (8%–27%) [8–10]. Previous studies confirmed the disparity in ICAS prevalence by race/region [11–15], but the epidemiological characteristics of ICAS located in anterior circulation (AC) and posterior circulation (PC) has been scarcely noted. In addition, the ICAS of AC and PC shared risk factors; for example, hypertension and diabetes are both the risk factors of ICAS [16, 17]. Different magnitudes of atherosclerotic lesion's risk for AC compared with PC were less reported in general population. These emerging risk factors associated with the presence of ICAS according to the anatomic location of intracranial artery (i.e., AC and PC as well as per-vessel) are not yet clarified.

Expanding our understanding of ICAS prevalence by the anatomic location of intracranial artery and the association between risk factors and ICAS by location will afford insight in the pathogenesis of ICAS. This would provide potential implications for precise ICAS prevention. We therefore aimed to fill this knowledge gap by conducting a study to determine ICAS prevalence of AC and PC in a representative population. We were also interested in determining if the profiles of risk factor differed by atherosclerotic location.

## 2. Materials and Methods

### 2.1. Survey Participants

Data was used from the Beijing subgroup of the China Hypertension Survey (CHS), a nation-wide survey performed during the period of 2012–2015. The study design and major findings of the survey had been published elsewhere [18, 19]. Briefly, a stratified multistage multicentered national cross-sectional survey was conducted to investigate the prevalence of hypertension in subjects over 15 years old from 31 metropolises/provinces in China. For this substudy, all selected urban and rural areas of 31 metropolises/provinces were stratified into eastern, middle and western regions again according to both geographical locations and economic level. Sixty cities and seventy counties were selected by using the simple random sampling (SRS) method. Beijing was one of the selected cities, and three districts and one county were randomly selected for the survey. Next, three communities or villages were further randomly selected. Only residents with certificated documents from the Administration of Households of the local government were enrolled to exclude immigration effects. According to the protocol of CHS, 4000 residents aged more than 35 years were the designed sample size. Taking nonresponse into account, an additional 20% sample was added, and thus, the final sample size was 4800 participants. All participants were invited to complete a standard questionnaire, blood biochemical testing, and cerebral vascular evaluation by carotid ultrasound and transcranial Doppler (TCD) as a secondary analysis for atherosclerosis. A total of 3954 participants completed the questionnaire and underwent the TCD vascular evaluation with a response rate of 82.4%; thus, they were included in this analysis. The participant screening flowchart was presented in Figure S1. The characteristics between participants included and not included in the analyses are listed in Table S1.

The protocol of this study's design was approved by the ethics committee of the Xuanwu Hospital of Capital Medical University ([2014]-016) and Fuwai Hospital (2012-402). Written informed consent was obtained from all participants before enrollment.

### 2.2. Training and Data Collection

A standardized questionnaire developed by Fuwai Hospital, the coordination center of this nation-wide survey, was administrated by a trained staff to collect information about demographic characteristics, lifestyle, risk factors, and history of coronary heart disease, stroke, diabetes, and hyperlipidemia [19]. The histories of coronary heart disease and stroke were self-reported and verified by cardiologists or neurologists, respectively. Blood pressure and body weight and height were measured with a standard procedure, and details were described elsewhere [18–20]. After the interview, participants were invited to take laboratory measurements. Blood samples were taken and sent to the National Center for Cardiovascular Disease of Fuwai Hospital for analysis according to predefined protocols to ensure the accuracy and controllability of the results [20].To ensure the quality of survey, all of the investigators received standard training before participation, including the TCD operators. All physical examinations and laboratory testing were conducted strictly as the protocol required.

### 2.3. Assessment of Intracranial Artery Stenosis

TCD was performed by two independent vascular ultrasound practitioners with more than five years of experience using portable machines. All procedures were carried out as the standard protocol required, and each vessel in the intracranial artery was detected, including the bilateral anterior cerebral artery (ACA), middle cerebral artery (MCA), posterior cerebral artery (PCA), terminal of internal carotid artery (ICA), vertebral artery (VA), and basilar artery (BA). The diagnoses of ICAS referred to Wong's criteria based on peak systolic flow velocity (Vp) [21]. The cutoff value of Vp for ICAS diagnosis was 140 cm/s in the MCA, 120 cm/s in the ACA and internal carotid siphon, and 100 cm/s in the PCA and vertebra-basilar artery. Additional criteria of stenosis in the MCA were as follows: Vp ranged from 140 to 160 cm/s, together with disturbance in echo frequency and turbulence, or Vp reduction by ≥30% compared with the contralateral depth-corresponding homologous segment. Intracranial artery occlusion is considered when low velocity and low resistance discontinuous flow signals are detected along the main intracranial artery. The primary TCD criteria at each site of occlusion was defined as one of four types, such as dampened signal, blunted signal, minimal signal, and absent signal [22]. Apart from the velocity criteria, the subjects' age was also considered. In the absence of good temporal windows, intracranial blood flow signals were detected via orbital window [21]. Any cerebral arteries that could not be detected via both temporal and orbital window were considered nonstenosis due to the failed detection of blood follow by both the temporal and orbital windows. The operators and reviewers of the TCD studies were blind to the clinical information.

### 2.4. Definition of Vascular Risk Factors

Hypertension was defined as systolic blood pressure ≥140 mmHg and/or diastolic blood pressure ≥90 mmHg or having antihypertension therapy. Diabetes was defined as having a previous history of diabetes or on insulin or oral hypoglycemic medication, or having a fast blood glucose over 7 mmol/L. Hyperlipidemia was defined as total cholesterol ≥6.1 mmol/L or triglyceride ≥2.26 mmol/L or high density lipoprotein cholesterol, <0.9 mmol/L in males and <1.0 mmol/L in female, or under lipid-lowering medication. Smoking was defined as those who were either ex-smokers or current smokers. Body mass index (BMI) was classified as underweight (<18.5), normal (18.5–24.9 kg/m^2^), overweight (25.0–29.9 kg/m^2^), and obese (>30.0 kg/m^2^) [18]. Hyperuricaemia was defined as a serum uric acid level ≥416 *μ*mol/L for male and ≥357 *μ*mol/L for female. Hyperhomocysteinemia was defined as serum homocysteine ≥15 umol/L, and urine microalbuminuria (UMA) was defined as urine microalbumin ≥20 mg/L.

### 2.5. Statistical Analysis

The CHS was designed to provide accurate estimates for the prevalence of cardiovascular disease and risk factors in the general Chinese population as well as for each selected metropolis/province by sampling weights calculated based on different population census data and sampling scheme and including oversampling for old age and nonresponses. In the current study, we estimated prevalence by age and gender based on the 2010 Beijing municipal population census. Therefore, we could provide prevalent estimates referable to the overall Beijing population. The age- and gender-specific weight-adjusted sample was acquired to estimate the prevalence of ICAS. The association of risk factors with the presence of ICAS was analyzed by ANOVA, student's *t*-test, and chi-squared test. To identify the potential risk factors independently associated with the presence of ICAS, multivariate logistic regression models were allied. Odds ratios (OR) and corresponding 95% confidence intervals (95% CI) were also estimated using multivariate logistic regression analysis.

## 3. Results

**Figure 1:**
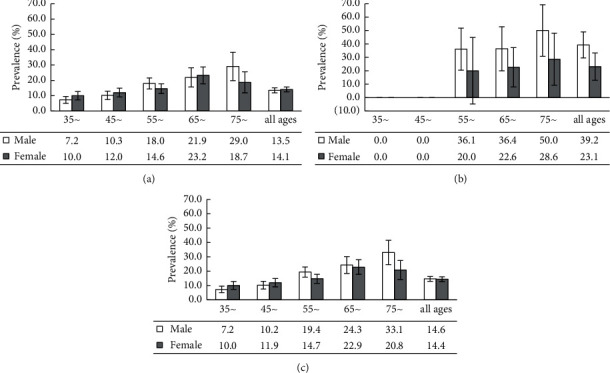
Prevalences and 95% CIs of ICAS by age and gender. (a) Population free of stroke; (b) population with history of stroke; (c) total population.

The results of Table 1 showed that the overall weighted prevalence of ICAS was 14.6% (95%CI: 13.5–15.8). The prevalence of ICAS was much lower in population free of stroke than that with a history of stroke (13.8% vs. 32.9%). The prevalence of males was similar to females (14.9% vs. 14.4%), but the increased magnitude across age groups was different by gender (Figure 1(a)), and the increased patterns were similar for population with and without history of stroke (Figures 1(b) and 1(c)).

Table 1 also demonstrates that the ICAS prevalence in AC was much higher than that in PC (11.9% vs. 4.2%). The vascular lesions in PC increased faster than in AC with advanced age, and this rapid increase was attributed mainly to VA lesions.

A total of 987 arteries were detected to have atherosclerotic lesions (892 stenosis and 45 occlusions) among 752 individuals with vascular lesions. The individuals with 1, 2, 3, 4, and 5 or more artery lesions were 302, 122, 46, 44, and 14, respectively.

Risk factors related to ICAS were the highest in the subjects with both ICAS and history of stroke (Table S2). The average age for individuals without ICAS was 61.4 ± 12.8 years old, followed by those with ICAS of AC (66.0 ± 11.2), and those with ICAS in PC had the highest age (69.8 ± 8.8). The frequency and multivariable analyses in the population free of stroke were showed in Tables S3 and 2. The odds of hypertension and diabetes increased by 79% (OR: 1.79, 95% CI: 1.40–2.27) and by 30% (OR: 1.35, 95% CI: 1.04–1.75) in those with AC lesions and by 3.35 times (OR: 3.35, 95% CI: 2.49–4.50) and 71% (OR: 1.71, 95% CI: 1.19–2.46) in those with PC lesions compared with that without ICAS. The odds of overweight or obese were reduced in those with either AC or PC lesions. In addition, the population with PC lesions was more likely to have diabetes, hyperlipidemia, and elevated high-sensitivity C-reactive protein (hi-CRP). Most OR values in PC lesions were greater than those in AC lesion, but all of 95% CIs were overlapped, except age.

The frequency and multivariable analyses in subjects with a history of stroke showed that males were more likely to have lesions in AC, and diabetes was significantly associated with lesions of PC (Tables S4 and S5).

Per-vessel risk factor analysis for subjects without and with a history of stroke is listed in Table S6. The associations of most of the previously mentioned risk factors with each intracranial artery lesion were similar to those with AC and PC lesions. However, in the subpopulation with stroke history, the odds of UMA in those with ICAS of VA were significantly higher compared to those with non-ICAS.

## 4. Discussion

In this representative general population study of Beijing, China, using TCD to detect the vascular lesions of the intracranial artery, we found that ICAS of AC was the predominant location. Our evidence suggested that most risk factors may differ, but there were no different magnitudes of risk for ICAS of AC compared with PC, except for age. On multivariable analyses, hypertension and diabetes were independent risk factors for ICAS of both AC and PC, while hyperlipidemia and elevated hi-CRP were independently associated with PC lesions. To the best of our knowledge, this is the first population-based study to estimate the prevalence of ICAS by the anatomical location of intracranial atherosclerosis. The large sample and randomly selected population of the current study allowed us to analyse the characteristics by age-gender subgroup. The method for detecting vascular lesions and the criteria for defining atherosclerosis were consistent with most of the previous studies in China [11, 21]. Our ICAS prevalence of 13.8% in the population free of stroke is similar to that in the APCA study [11]. The APAC study was performed on 5353 employees aged 40 years or older and retirees of a large coal mine industry in Kailuan, located 150 km southeast of our study site.

Arteries located in AC were more likely to experience atherosclerosis than those in PC. The finding corresponded with the previous clinical observation studies [7, 11, 23, 24]. About 70–80% of the patients who experienced ischemic stroke had AC lesions, and MCA was the most common affected artery. In the current study, we also found that the atherosclerotic lesion was high in MCA. We also analyzed PC lesions and found that VA was much more likely to have atherosclerotic lesions. Several population-based studies of China [11], Japan [25], Korea [13], and Spain [15] demonstrated that MCA was much more likely to have atherosclerotic lesions. Similar findings were also noted in hospitalized ischemic stroke patients in China [3] and European countries such as Italy [26] and Germany [9]. However, the prevalence of each intracranial artery was not reported in these studies. In an autopsy study in France, Mazighi et al. reported that MCA and BA appeared to be the most common location for stenosis >30%. Similarly, Kimura et al. reported that MCA and BA had the highest percentage of severe atherosclerosis from 7260 autopsies in Japan, but the lesions in ACA was the lowest. On the contrary, Suri et al. reported that intracranial artery lesions in PCA were highest in the Atherosclerosis Risk in Community Study of US [27]. Whether the diversity in the location or artery of atherosclerotic lesions among these previous studies was due to the differences in study design, method for detecting vascular lesion, definition of ICAS, or selection of the study population was not clear, and thus, the results should be explained with caution.

We found that the association of demographic characteristics to vascular lesions by location was interesting. The mean age of the subjects with AC lesions was 3.9 years younger than those with PC lesions. The findings are in accordance with hospital-based studies in China [23, 28, 29]. Compared with patients with PC stroke, the patients with AC stroke were 0.9–5.6 years younger. On the contrary, studies from Western countries such as Switzerland, Germany, Austria, and Czech showed that patients with posterior circulation infarction were 3–5 years younger than those with anterior circulation infarction [30–33]. The earlier occurrence of ICAS in AC in the current study can well explain why the age at the first stroke onset in China was about 10 years younger than that in the Western population [34]. However, we did not find that ICAS had sex differences in our population despite the fact that stroke incidence is generally higher in males than females [1]. The average age of females with ICAS was younger than males, which suggested that vascular lesions occur earlier in females. But age-specific prevalence suggested that the vascular lesion might develop faster in males than females, which consequently led to the higher ICAS prevalence in males than females after the age of 55 years. This gender–age change pattern was similar to the Asymptomatic Polyvascular Abnormalities Community (APCA) Study [11]. We could not explain the earlier onset and the slower progression of vascular lesions in females compared with males. It is known that the risk of stroke and majority of risk factors in women are closer to those in men after menopause [35]. Female sex hormones may be implicated in vascular outcomes [35].One possible explanation might be that unhealthy lifestyles (i.e., smoking and physical inactivity) are more prevalent in men than women across the life span.

The modifiable risk factors associated with ICAS in current study were much similar to a previous Chinese population-based study [11]. Hypertension, diabetes, and elevated hi-CRP were independent risk factors for ICAS [36], while overweight and obesity appeared to have a protective effect against vascular damage [11]. We failed to find any unique potential risk factor that can explain the high prevalence of ICAS in our population. It is known that the prevalence of smoking [37] and hyperhomocysteinemia [38] are generally higher in Chinese population than the Western population. However, we did not find the significant differences of these two risk factors between individuals with and without ICAS. Perhaps, some unmeasured characteristics associated with the development of ICAS may explain the high incidence of ICAS in the Asian population and need to be elucidated in the future.

Our analysis showed that the modifiable risk factors in lesions between AC and PC were similar, except for age, which is not in line with the previous studies [2, 11–15]. To date, there has been no study attempting to discern the comparative role of these risk factors by the anatomic location of intracranial artery lesions in general population. Several hospital-based studies showed the disparity of risk factor profile in ischemic stroke patients located in AC and PC existed. Compared with patients with AC ischemic stroke, patients with PC ischemic stroke had 50% higher prevalence of diabetes and 16% higher prevalence of hypertension, and TG was high, while HDL-C was lower in Chinese ischemic stroke patients [28]. Subramanian et al. found that diabetes was independently associated with an increase in the odds of PC to AC ischemic stroke in 849 Canadian patients with acute ischemic stroke [39]. The authors presumed a neurovascular origin of anterior and posterior circulation differed and the arteries of PC might be more susceptible to metabolic disorders. We did not find the difference in these modifiable risk factors in the population with history of stroke. The values of ORs were greater than these of AC, but all of 95% CIs were overlapped. Our findings implied that an intensive administration of those modifiable vascular risk factors in the population free of stroke and that with a history of stroke could reduce of the risk of ICAS; hence, risk of the first-ever and recurrence of stroke would decrease. Controlling diabetes at population level might decrease the risk of ICAS in PC, which may reduce the risk of PC strokes and generally have worse outcomes compared with AC strokes [28].

Limitations of the current study have to be mentioned. First, the ICAS assessment was by TCD and was not further validated by vessel imaging (CTA or MRA). The application of the golden standard measurements, that is, DSA, CTA, or MRA, is not feasible in large population studies. TCD was applied to detect intracranial artery lesions in most population-based surveys in China [11, 21, 40], and the sensitivity was validated by a previous study [41, 42]. With an identical criterion to define vascular lesions, our results were comparable with those of previous studies [11, 21, 40]. Second, subjects had an acoustic window failure, and they were defined as non-ICAS, which would lead to misclassification of ICAS and underestimation of ICAS burdens. We failed to detect blood flow using the temporal or orbital windows in only 34 subjects. Therefore, the impact on the prevalence of CAS was not big enough to change the overall prevalence. Third, the cross-sectional design of this study limits the ability to draw definitive associations of risk factors with the development of ICAS. We will follow this population for several years, so the causal relationship between the development of ICAS and risk factors can be explored.

In conclusion, our results showed that the prevalence of ICAS in AC was predominate to that in PC. The vascular lesion in PC developed faster than that in AC with aging. Advanced age in patients with ICAS appeared to exert different magnitudes of risk for PC to AC lesions. The ICAS of AC and PC shared the most modifiable risk factors.

## Figures and Tables

**Table 1:** Weighted prevalent rates and 95% CIs of ICAS by the anatomic location.

Age	Prevalence and 95% CI of anterior circulation				Prevalence and 95% CI of posterior circulation			
	Total	Subtotal	ACA	MCA	Subtotal	PCA	VA	BA
*Population free of stroke*								
35∼	8.6 (6.9∼10.3)	6.9 (5.3∼8.4)	5.4 (4.0∼6.9)	3.3 (2.2∼4.4)	2.4 (1.4∼3.4)	0.2 (0.0∼0.5)	2.2 (1.3∼3.1)	—
45∼	10.9 (8.9∼12.9)	10.0 (8.1∼11.9)	7.2 (5.5∼8.9)	5.7 (4.2∼7.2)	1.6 (0.8∼2.5)	0.2 (0.0∼0.6)	0.9 (0.3∼1.6)	0.6 (0.1∼1.1)
55∼	16.8 (14.3∼19.3)	15.5 (13.1∼17.9)	10.2 (8.1∼12.3)	10.7 (8.5∼12.8)	4.9 (3.4∼6.4)	1.9 (0.9∼2.8)	2.9 (1.7∼4.1)	0.7 (0.1∼1.3)
65∼	22.8 (18.7∼27.0)	19.1 (15.2∼23.1)	12.4 (8.9∼15.9)	13.1 (9.6∼16.7)	10.1 (6.9∼13.3)	2.3 (0.6∼3.9)	7.9 (5.0∼10.8)	2.9 (1.0∼4.7)
75∼	23.1 (17.3∼29.0)	18.2 (12.7∼23.7)	10.5 (5.9∼15.1)	13.6 (8.5∼18.6)	12.1 (7.2∼16.9)	2.5 (0.1∼5.0)	9.5 (5.1∼13.9)	3.2 (0.4∼5.9)
Total	13.8 (12.6∼14.9)	11.9 (10.8∼13.0)	8.2 (7.2∼9.1)	7.5 (6.6∼8.4)	4.2 (3.5∼4.9)	1.0 (0.6∼1.3)	3.0 (2.4∼3.6)	0.8 (0.5∼1.1)
*P* for trend	<0.0001	<0.0001	<0.0001	<0.0001	<0.0001	<0.0001	<0.0001	<0.0001

*Population with history of stroke*								
35∼	—	—	—	—	—	—	—	—
45∼	—	—	—	—	—	—	—	—
55∼	32.7 (19.5∼45.8)	31.3 (18.1∼44.4)	25.0 (12.2∼37.8)	19.5 (7.4∼31.6)	21.4 (9.0∼33.8)	13.2 (2.4∼23.9)	17.5 (5.7∼29.3)	5.7 (0.0∼13.4)
65∼	32.3 (20.9∼43.7)	25.4 (14.3∼36.5)	17.0 (6.9∼27.1)	18.5 (8.2∼28.9)	22.8 (11.9∼33.7)	10.2 (1.7∼18.7)	17.0 (6.9∼27.1)	8.3 (0.5∼16.2)
75∼	38.1 (23.4∼52.8)	27.8 (13.1∼42.4)	21.2 (7.3∼35.2)	21.2 (7.3∼35.2)	29.7 (15.0∼44.5)	7.1 (0.0∼16.7)	25.7 (11.2∼40.2)	10.3 (0.0∼21.4)
Total	32.9 (25.7∼40.2)	27.0 (19.9∼34.2)	20.0 (13.3∼26.7)	18.8 (12.2∼25.4)	23.4 (16.4∼30.4)	10.0 (4.6∼15.4)	18.8 (12.2∼25.4)	7.7 (2.9∼12.5)
*P* for trend	<0.0001	0.198	0.959	0.729	0.189	0.715	0.203	0.137

*Overall population*								
35∼	8.6 (6.9∼10.3)	6.9 (5.3∼8.4)	5.4 (4.0∼6.9)	3.3 (2.2∼4.4)	2.4 (1.4∼3.4)	0.2 (−0.1∼0.5)	2.2 (1.3∼3.1)	
45∼	10.9 (8.9∼12.8)	9.9 (8.0∼11.8)	7.2 (5.5∼8.9)	5.6 (4.1∼7.1)	1.6 (0.8∼2.5)	0.2 (−0.1∼0.6)	0.9 (0.3∼1.6)	0.6 (0.1∼1.1)
55∼	17.6 (15.2∼20.1)	16.2 (13.8∼18.6)	10.9 (8.8∼13.0)	11.1 (9.0∼13.2)	5.7 (4.2∼7.3)	2.4 (1.3∼3.5)	3.6 (2.3∼4.9)	0.9 (0.2∼1.6)
65∼	24.2 (20.3∼28.1)	20.0 (16.2∼23.7)	12.8 (9.5∼16.0)	13.6 (10.3∼16.9)	11.9 (8.7∼15.1)	3.3 (1.5∼5.1)	8.7 (5.9∼11.5)	3.5 (1.6∼5.4)
75∼	25.7 (20.2∼31.2)	19.7 (14.5∼25.0)	12.0 (7.6∼16.4)	14.1 (9.4∼18.8)	15.2 (10.3∼20.0)	3.2 (0.7∼5.7)	11.6 (7.2∼16.0)	4.2 (1.3∼7.0)
Total	14.6 (13.5∼15.8)	12.5 (11.5∼13.6)	8.6 (7.7∼9.5)	7.9 (7.0∼8.8)	5.0 (4.3∼5.8)	1.3 (0.9∼1.7)	3.6 (3.0∼4.2)	1.1 (0.7∼1.4)
*P* for trend	<0.0001	<0.0001	<0.0001	<0.0001	<0.0001	<0.0001	<0.0001	<0.0001

**Table 2:** ORs and 95% CIs of ICAS for 3600 individuals free of stroke^*∗*^.

Risk factors	Anterior circulation		Posterior circulation	
	Crude OR and 95% CI	Fully adjusted OR and 95% CI	Crude OR and 95% CI	Fully adjusted OR and 95% CI
Age (<65 vs. ≥65 years old)	1.79 (1.48∼2.17)	1.18 (0.93∼1.47)	3.37 (2.46∼4.61)	2.16 (1.48∼3.15)
Gender (male vs. female)	0.89 (0.73∼1.07)	0.88 (0.66∼1.17)	1.07 (0.80∼1.43)	1.04 (0.68∼1.61)
Hypertension (no vs. yes)	1.99 (1.65∼2.41)	1.79 (1.40∼2.27)	3.35 (2.49∼4.50)	2.54 (1.76∼3.69)
Diabetes (no vs. yes)	1.76 (1.42∼2.17)	1.35 (1.04∼1.75)	2.42 (1.80∼3.25)	1.71 (1.19∼2.46)
Hyperlipidemia (no vs. yes)	1.12 (0.93∼1.36)	1.05 (0.83∼1.32)	1.52 (1.13∼2.04)	1.49 (1.04∼2.16)
History of CAD (no vs. yes)	2.00 (1.32∼3.01)	1.23 (0.71∼2.12)	3.72 (2.30∼6.00)	1.86 (0.97∼3.56)
*Smoking status*				
Never	Reference	Reference	Reference	Reference
Current smoker	1.06 (0.84∼1.34)	1.00 (0.71∼1.39)	0.97 (0.68∼1.39)	1.25 (0.75∼2.08)
Ex-smoker	1.38 (1.02∼1.88)	1.22 (0.81∼1.83)	1.19 (0.74∼1.91)	1.02 (0.54∼1.92)
Normal weight	Reference	Reference	Reference	Reference
Underweight	0.83 (0.35∼1.97)	1.19 (0.45∼3.13)	0.61 (0.15∼2.57)	0.96 (0.22∼4.20)
Overweight	1.01 (0.83∼1.23)	0.93 (0.73∼1.18)	0.82 (0.61∼1.11)	0.61 (0.42∼0.88)
Obese	0.87 (0.63∼1.20)	0.61 (0.40∼0.92)	0.69 (0.42∼1.15)	0.33 (0.17∼0.65)
Hyperuricaemia (no vs. yes)	1.31 (1.02∼1.68)	1.17 (0.87∼1.58)	1.65 (1.17∼2.33)	1.07 (0.69∼1.67)
Hyperhomocysteinemia (no vs. yes)	0.99 (0.80∼1.22)	0.94 (0.74∼1.18)	1.04 (0.76∼1.44)	0.94 (0.66∼1.34)
hi-CRP(mg/dl)				
1st tertile (≤0.70)	Reference	Reference	Reference	Reference
2nd tertile (0.71–1.80)	0.77 (0.61∼0.98)	1.02 (0.77∼1.35)	0.84 (0.58∼1.20)	1.07 (0.68∼1.67)
3rd tertile (>1.80)	0.98 (0.78∼1.22)	1.27 (0.97∼1.67)	1.37 (1.00∼1.89)	1.69 (1.12∼2.56)
UMA (no vs. yes)	1.21 (0.99∼1.47)	1.27 (0.97∼1.67)	1.59 (1.19∼2.12)	1.23 (0.87∼1.75)

^*∗*^Population with neither ICAS nor stroke was the reference group, and the frequencies of risk factors are listed in Supplementary Table S3.

## Data Availability

The data that support the findings of this study are available from the corresponding author upon reasonable request.

## Supplementary Materials

Supplementary Materials Supplementary Figure 1: the screening flowchart of participants. Supplementary Table 1: characteristics (i.e., age, sex, smoking and alcohol consumption, and chronic diseases). Supplementary Tables 2–4: frequency of risk factors (i.e., old age, male, hypertension and history of coronary heart disease, smoking, obesity, and laboratory testing results) in the population with or without history of stroke by anatomic location of ICAS. Supplementary Tables 5–6: ORs and 95% CIs of ICAS in the intracranial anterior and posterior circulation and branches for individuals with or without history of stroke.
