# Supplementary material for: Prevalence and Risk Factors Comparison of Anterior and Posterior Intracranial Arterial Stenosis
Source: Evid Based Complement Alternat Med. 2022 Jan 10;2022:7710374. doi: 10.1155/2022/7710374 (PMC8763509; doi:10.1155/2022/7710374)
Supplement: Supplementary Materials — Supplementary Figure 1: the screening flowchart of participants. Supplementary Table 1: characteristics (i.e., age, sex, smoking and alcohol consumption, and chronic diseases). Supplementary Tables 2–4: frequency of risk factors (i.e., old age, male, hypertension and history of coronary heart disease, smoking, obesity, and laboratory testing results) in the population with or without history of stroke by anatomic location of ICAS. Supplementary Tables 5–6: ORs and 95% CIs of ICAS in the intracranial anterior and posterior circulation and branches for individuals with or without history of stroke. [file 7710374.f1.docx]

Participants completed baseline questionnaires

(n=4795)

Participants unable to complete TCD examination were excluded. (n=841)

Participants completed TCD examination

(n=3954)

Population with history of stroke(n=354)

Population free of stroke

(n=3600)

No-ICAS (n=120)

No-ICAS (n=234)

ICAS

(n=632)

No-ICAS (n=2968)

Supplementary Figure 1. Participant screening flow chart

TCD:Trancranial Doppler

ICAS: Intracranial Arterial Stenosis

Supplementary Table 1. Characteristics of individuals underwent vs those not underwent TCD assessment

| Characteristics | Not underwent TCD assessment | Underwent TCD assessment | P value |
| --- | --- | --- | --- |
| N | 841 | 3954 |  |
| Age ±SD (year) | 59.1±15.0 | 62.45±12.7 | <0.001 |
| Male,%(n) | 47.6(400) | 42.8(1692) | <0.001 |
| Overweight or obese, %(n) | 52.2(439) | 55.1(1305) | 0.280 |
| Current smoker, %(n) | 21..6(182) | 20.1(791) | 0.233 |
| Ex-smoker, %(n) | 8.3(70) | 10.0(395) | 0.260 |
| Hypertension, %(n) | 43.8(369) | 58.2(2300) | <0.001 |
| Diabetes, %(n) | 15.6(131) | 23.3(923) | <0.001 |
| History of coronary heart events, %(n) | 3.0(25) | 4.6(183) | 0.032 |
| History of stroke | 7.0(58) | 8.9(354) | 0.185 |
| Hyperlipidemia, %(n) | 43.9(693) | 59.1(2336) | <0.001 |

Supplementary Table 2. Frequency of risk factors in the population free of stroke and with history of stroke by ICAS

|  | Population free of stroke | | |  | Population with history of stroke | | |
| --- | --- | --- | --- | --- | --- | --- | --- |
| Risk factors | ICAS- (n=2968) | ICAS+ (n=632) | P value |  | ICAS- n=234) | ICAS+ (n=120) | P value |
| ≥65 years old, %(n) | 44.3(1316) | 61.7(390) | <0.001 |  | 85.0(199) | 88.3(106) | 0.723 |
| Male, %(n) | 41.2(1222) | 43.8(277) | 0.920 |  | 49.1(115) | 66.7(80) | 0.038 |
| Hypertension, %(n) | 37.4(1110) | 55.7(352) | <0.001 |  | 75.6(177) | 74.2(89) | 0.837 |
| Diabetes, %(n) | 19.3(573) | 30.9(195) | <0.001 |  | 31.6(74) | 45.0(54) | 0.051 |
| Hyperlipidemia, %(n) | 56.4(1673) | 59.7(377) | 0.274 |  | 64.5(151) | 56.7(68) | 0.109 |
| History of CAD, %(n) | 3.2(95) | 6.8(43) | 0.004 |  | 10.3(24) | 14.2(17) | 0.785 |
| Smoking status |  |  |  |  |  |  |  |
| Never smoker, %(n) | 71.2(2112) | 69.0(436) | 0.203 |  | 65.8(154) | 54.2(65) | 0.304 |
| current smoker, %(n) | 20.4(604) | 20.6(130) |  |  | 15.4(36) | 18.3(22) |  |
| ex-smoker, %(n) | 8.5(252) | 10.4(66) |  |  | 18.8(44) | 27.5(33) |  |
| BMI (kg/㎡) |  |  |  |  |  |  |  |
| Normal weight, %(n) | 47.9(1421) | 49.5(313) | 0.687 |  | 46.6(109) | 42.5(51) | 0.745 |
| Underweight, %(n) | 1.4(41) | 0.9(6) |  |  | 2.1(5) | 3.3(4) |  |
| Overweight, %(n) | 39.2(1162) | 40.2(254) |  |  | 36.3(85) | 43.3(52) |  |
| Obese, %(n) | 11.6(344) | 9.3(59) |  |  | 15.0(35) | 10.8(13) |  |
| Hyperuricaemia, %(n) | 14.2(421) | 18.4(116) | 0.121 |  | 20.5(48) | 20.0(24) | 0.829 |
| Hyperhomocysteinemia | 53.3(1363) | 52.0(248) | 0.005 |  | 55.1(102) | 62.9(56) | 0.723 |
| n=3306, %(n) |  |  |  |  |  |  |  |
| Hi-CRP(mg/dl) |  |  |  |  |  |  |  |
| 1st tertile(≤0.70), %(n) | 46.1(1369) | 47.8(302) | 0.313 |  | 39.7(93) | 40.0(48) | 1.000 |
| 2nd tertile, %(n) | 27.6(818) | 23.1(146) |  |  | 32.1(75) | 25.0(30) |  |
| 3rd tertile, %(n) | 26.3(781) | 29.1(184) |  |  | 28.2(66) | 35.0(42) |  |
| UMA, %(n) n=3680, %(n) | 36.7(1014) | 41.7(246) | 0.094 |  | 45.5(100) | 48.2(53) | 0.220 |

Supplementary Table 3. Frequency of risk factors in the population free of stroke by anatomic location of ICAS

| Risk factors | Non-ICAS | ICAS in anterior circulation | P value* | ICAS in posterior circulation | P value** |
| --- | --- | --- | --- | --- | --- |
| ≥65 years old, %(n) | 44.3(1316) | 58.8(304) | <0.001 | 72.9(153) | <0.001 |
| Male, %(n) | 41.2(1222) | 44.1(228) | 0.213 | 39.5(83) | 0.639 |
| Hypertension, %(n) | 37.4(1110) | 54.4(281) | <0.001 | 66.7(140) | <0.001 |
| Diabetes, %(n) | 19.3(573) | 29.6(153) | <0.001 | 36.7(77) | <0.001 |
| Hyperlipidemia, %(n) | 56.4(1673) | 59.2(306) | 0.232 | 66.2(139) | 0.005 |
| History of CAD, %(n) | 3.2(95) | 6.2(32) | 0.001 | 11.0(23) | <0.001 |
| Smoking status |  |  |  |  |  |
| Never smoker, %(n) | 71.2(2112) | 68.1(352) | 0.117 | 70.5(148) | 0.741 |
| Current smoker, %(n) | 20.4(604) | 20.7(107) |  | 19.5(41) |  |
| Ex-smoker, %(n) | 8.5(252) | 11.2(58) |  | 10.0(21) |  |
| Normal weight | 47.9(1421) | 48.5(251) | 0.806 | 53.8(113) | 0.355 |
| Underweight, %(n) | 1.4(41) | 1.2(6) |  | 1.0(2) |  |
| Overweight, %(n) | 39.2(1162) | 40.0(207) |  | 36.2(76) |  |
| Obese, %(n) | 11.6(344) | 10.3(53) |  | 9.0(19) |  |
| Hyperuricaemia, %(n) | 14.2(421) | 17.8(92) | 0.033 | 21.4(45) | 0.004 |
| Hyperhomocysteinemia, %(n) | 53.3(1363) | 53.0(204) | 0.895 | 54.4(86) | 0.791 |
| Hi-CRP(mg/dl) |  |  |  |  |  |
| 1st tertile(≤0.70, %(n) | 46.1(1369) | 49.5(256) | 0.079 | 43.8(92) | 0.028 |
| 2nd tertile, %(n) | 27.6(818) | 22.8(118) |  | 21.9(46) |  |
| 3rd tertile, %(n) | 26.3(781) | 27.7(143) |  | 34.3(72) |  |
| UMA, %(n) | 36.7(1014) | 41.3(198) | 0.059 | 45.2(95) | 0.002 |

* non-ICAS vs ICAS in anterior circulation; ** non-ICAS vs ICAS in posterior circulation;

Supplementary Table 4. Frequency of risk factors in the population with history of stroke by anatomic location of ICAS

| Risk factors | Non-ICAS | ICAS in Anterior circulation | P value* | ICAS in posterior circulation | P value** |
| --- | --- | --- | --- | --- | --- |
| ≥65 years old, %(n) | 85.0(199) | 84.9(73) | 0.972 | 89.7(70) | 0.297 |
| Male, %(n) | 49.1(115) | 73.3(63) | <0.001 | 67.9(53) | 0.004 |
| Hypertension, %(n) | 75.6(177) | 75.6(65) | 0.991 | 73.1(57) | 0.651 |
| Diabetes, %(n) | 31.6(74) | 45.3(39) | 0.023 | 50.0(39) | 0.003 |
| Hyperlipidemia, %(n) | 64.5(151) | 55.8(48) | 0.154 | 61.5(48) | 0.684 |
| History of CAD, %(n) | 10.3(24) | 14.0(12) | 0.353 | 17.9(14) | 0.072 |
| Smoking status |  |  |  |  |  |
| Never smoker, %(n) | 65.8(154) | 50.0(43) | 0.03 | 52.6(41) | 0.099 |
| Current smoker, %(n) | 15.4(36) | 19.8(17) |  | 19.2(15) |  |
| Ex-smoker, %(n) | 18.8(44) | 30.2(26) |  | 28.2(22) |  |
| BMI (kg/㎡) |  |  |  |  |  |
| Normal weight, %(n) | 46.6(109) | 39.5(34) | 0.297 | 47.7(37) | 0.428 |
| Underweight, %(n) | 2.1(5) | 2.3(2) |  | 5.1(4) |  |
| Overweight, %(n) | 36.3(85) | 47.7(41) |  | 37.2(29) |  |
| Obese, %(n) | 15.0(35) | 10.5(9) |  | 10.3(8) |  |
| Hyperuricaemia, %(n) | 20.5(48) | 19.8(17) | 0.883 | 20.5(16) | 1 |
| Hyperhomocysteinemia, %(n) | 55.1(102) | 63.6(42) | 0.231 | 63.8(37) | 0.445 |
| Hi-CRP(mg/dl) |  |  |  |  |  |
| 1st tertile(≤0.70, %(n) | 39.7(93) | 38.4(33) | 0.462 | 39.7(31) | 0.317 |
| 2nd tertile, %(n) | 32.1(75) | 26.7(23) |  | 24.4(19) |  |
| 3rd tertile, %(n) | 28.2(66) | 34.9(30) |  | 35.9(28) |  |
| UMA, %(n) | 45.5(100) | 52.6(41) | 0.28 | 51.4(37) | 0.381 |

* non-ICAS vs ICAS in anterior circulation; ** non-ICAS vs ICAS in posterior circulation;

Supplementary Table 5. ORs and 95% CIs of ICAS for 354 individuals with history of stroke

| Risk factors | Anterior circulation | |  | Posterior circulation | |
| --- | --- | --- | --- | --- | --- |
|  | Crude OR and 95% CI | Fully adjusted OR and 95% CI |  | Crude OR and 95% CI | Fully adjusted OR and 95% CI |
| Age(<65 vs ≥65 years old) | 0.99(0.49～1.97) | 0.64(0.28～1.45) |  | 1.54(0.68～3.48) | 1.35(0.51～3.57) |
| Gender(male vs female ) | 0.35(0.21～0.61) | 0.38(0.17～0.84) |  | 0.46(0.27～0.78) | 0.51(0.22～1.16) |
| Hypertension(no vs yes ) | 1.00(0.56～1.77) | 0.95(0.46～1.94) |  | 0.87(0.49～1.57) | 1.13(0.52～2.45) |
| Diabetes(no vs yes ) | 1.79(1.08～2.98) | 1.84(0.93～3.63) |  | 2.16(1.28～3.65) | 2.16(1.11～4.20) |
| Hyperlipidemia(no vs yes ) | 0.69(0.42～1.15) | 0.60(0.31～1.17) |  | 0.88(0.52～1.49) | 0.96(0.48～1.95) |
| history of CAD (no vs yes ) | 1.42(0.68～2.98) | 0.84(0.31～2.34) |  | 1.91(0.94～3.92) | 1.06(0.40～2.79) |
| Smoking status |  |  |  |  |  |
| Never | Reference | Reference |  | Reference | Reference |
| Current smoker | 1.69(0.87～3.30) | 0.73(0.30～1.80) |  | 1.57(0.78～3.13) | 0.95(0.36～2.50) |
| Ex-smoker | 2.12(1.17～3.82) | 0.89(0.37～2.14) |  | 1.88(1.01～3.48) | 0.93(0.36～2.39) |
| Normal weight | Reference | Reference |  | Reference | Reference |
| Underweight | 1.28(0.24～6.91) | 1.29(0.11～14.95) |  | 2.36(0.60～9.24) | 1.81(0.26～12.57) |
| Overweight | 1.55(0.90～2.64) | 1.41(0.73～2.74) |  | 1.01(0.57～1.76) | 0.75(0.37～1.54) |
| Obese | 0.82(0.36～1.89) | 0.54(0.16～1.82) |  | 0.67(0.29～1.58) | 0.55(0.18～1.71) |
| Hyperuricaemia(no vs yes ) | 0.95(0.51～1.77) | 1.30(0.61～2.78) |  | 1.00(0.53～1.89) | 1.09(0.50～2.39) |
| Hyperhomocysteinemia(no vs yes) | 1.42(0.80～2.54) | 1.20(0.60～2.38) |  | 1.43(0.78～2.64) | 1.28(0.64～2.58) |
| Hi-CRP(mg/dl) |  |  |  |  |  |
| 1^st^ tertile(≤0.70) | 1 Reference | Reference |  | Reference | Reference |
| 2^nd^ tertile(0.71-1.80) | 0.86(0.47～1.60) | 1.31(0.54～3.15) |  | 0.76(0.40～1.45) | 1.05(0.43～2.52) |
| 3^rd^ tertile(>1.80) | 1.28(0.71～2.30) | 2.19(0.90～5.31) |  | 1.27(0.70～2.32) | 2.13(0.89～5.07) |
| UMA(no vs yes ) | 1.33(0.79～2.23) | 1.35(0.70～2.63) |  | 1.27(0.74～2.16) | 1.32(0.67～2.58) |

* population free of ICAS, but with history of stroke was the reference group and the frequencies of risk factors were listed in Supplementary Table 4.

Supplementary Table 6. ORs and 95% CIs of ICAS in each intracranial artery for individuals free and with history of stroke

| Subgroup | Free of stroke* | |  | With history of stroke** | |
| --- | --- | --- | --- | --- | --- |
|  | ACA(n=319) | |  | ACA(n=53) | |
| Risk factor | Crude OR and 95% CI | Fully adjusted OR and 95% CI |  | Crude OR and 95% CI | Fully adjusted OR and 95% CI |
| ≥65 years old | 1.56(1.24～1.97) | 1.06(0.79～1.42) |  | 0.86(0.39～1.92) | 0.62(0.24～1.62) |
| Male | 0.78(0.62～0.98) | 0.67(0.48～0.95) |  | 0.31(0.16～0.62) | 0.31(0.12～0.82) |
| Hypertension | 1.86(1.48～2.35) | 1.78(1.32～2.40) |  | 1.23(0.59～2.55) | 1.13(0.47～2.73) |
| Diabetes | 1.85(1.44～2.39) | 1.37(1.00～1.89) |  | 2.08(1.14～3.81) | 2.27(1.02～5.03) |
| Hyperlipidemia | 1.13(0.89～1.42) | 1.04(0.79～1.38) |  | 0.91(0.49～1.68) | 0.81(0.37～1.77) |
| History of CAD | 1.81(1.08～3.03) | 1.01(0.49～2.08) |  | 1.33(0.54～3.28) | 0.86(0.26～2.81) |
| Smoking status |  |  |  |  |  |
| Never smoker | Reference | Reference |  | Reference | Reference |
| Current smoker | 1.12(0.84～1.48) | 0.91(0.61～1.36) |  | 1.71(0.75～3.88) | 1.06(0.38～2.94) |
| Ex-smoker | 1.15(0.77～1.72) | 0.73(0.42～1.28) |  | 2.52(1.26～5.04) | 0.81(0.29～2.28) |
| BMI (kg/㎡) |  |  |  |  |  |
| Normal weight | Reference | Reference |  | Reference | Reference |
| Underweight | 0.83(0.29～2.33) | 1.42(0.49～4.15) |  | 1.82(0.33～9.93) | 2.46(0.21～29.50) |
| Overweight | 0.86(0.67～1.10) | 0.79(0.59～1.07) |  | 1.23(0.65～2.33) | 0.87(0.39～1.92) |
| Obese | 0.71(0.47～1.08) | 0.55(0.33～0.92) |  | 0.52(0.17～1.60) | 0.52(0.13～2.06) |
| Hyperuricaemia | 1.23(0.90～1.68) | 1.19(0.82～1.73) |  | 0.90(0.42～1.92) | 0.88(0.35～2.22) |
| Hyperhomocysteinemia | 0.87(0.67～1.14) | 0.77(0.58～1.03) |  | 1.27(0.64～2.54) | 1.12(0.50～2.54) |
| Hi-CRP(mg/dl) |  |  |  |  |  |
| 1st tertile(≤0.70 | Reference | Reference |  | Reference | Reference |
| 2nd tertile | 0.75(0.56～1.01) | 0.93(0.66～1.31) |  | 1.17(0.58～2.40) | 2.22(0.77～6.40) |
| 3rd tertile | 1.00(0.76～1.31) | 1.25(0.90～1.75) |  | 1.19(0.57～2.48) | 2.61(0.88～7.76) |
| UMA | 1.14(0.89～1.46) | 1.11(0.83～1.48) |  | 1.53(0.82～2.83) | 1.58(0.73～3.44) |
|  | MCA(n=321) | |  | MCA(n=57) | |
| ≥65 years old | 1.97(1.55～2.49) | 1.26(0.94～1.70) |  | 1.08(0.47～2.47) | 0.75(0.28～2.00) |
| Male | 0.90(0.72～1.14) | 0.87(0.61～1.24) |  | 0.31(0.16～0.61) | 0.40(0.15～1.06) |
| Hypertension | 2.28(1.80～2.88) | 2.03(1.51～2.74) |  | 1.35(0.65～2.77) | 1.26(0.52～3.05) |
| Diabetes | 1.92(1.49～2.47) | 1.35(0.98～1.85) |  | 1.69(0.94～3.05) | 1.48(0.66～3.32) |
| Hyperlipidemia | 1.25(0.98～1.58) | 1.22(0.92～1.63) |  | 0.53(0.30～0.95) | 0.45(0.21～0.98) |
| History of CAD | 2.23(1.38～3.59) | 1.23(0.65～2.35) |  | 1.43(0.61～3.37) | 0.55(0.14～2.12) |
| Smoking status |  |  |  |  |  |
| Never smoker | Reference | Reference |  | Reference | Reference |
| Current smoker | 1.03(0.77～1.38) | 0.99(0.66～1.50) |  | 2.14(1.00～4.57) | 1.04(0.37～2.92) |
| Ex-smoker | 1.37(0.94～2.00) | 1.19(0.73～1.95) |  | 2.42(1.22～4.82) | 0.97(0.35～2.73) |
| BMI (kg/㎡) |  |  |  |  |  |
| Normal weight | Reference | Reference |  | Reference | Reference |
| Underweight | 0.68(0.21～2.22) | 0.77(0.18～3.32) |  | - | - |
| Overweight | 1.08(0.85～1.38) | 0.95(0.71～1.28) |  | 1.69(0.91～3.15) | 1.27(0.58～2.78) |
| Obese | 0.81(0.54～1.22) | 0.51(0.30～0.87) |  | 0.71(0.25～2.01) | 0.46(0.09～2.35) |
| Hyperuricaemia | 1.31(0.96～1.77) | 1.15(0.80～1.65) |  | 0.54(0.23～1.27) | 0.81(0.30～2.17) |
| Hyperhomocysteinemia | 1.04(0.80～1.36) | 0.99(0.75～1.32) |  | 1.52(0.76～3.03) | 1.28(0.57～2.88) |
| Hi-CRP(mg/dl) |  |  |  |  |  |
| 1st tertile(≤0.70 | Reference | Reference |  | Reference | Reference |
| 2nd tertile | 0.89(0.67～1.18) | 1.15(0.81～1.62) |  | 0.72(0.35～1.49) | 0.94(0.34～2.62) |
| 3rd tertile | 1.04(0.79～1.38) | 1.35(0.96～1.91) |  | 1.12(0.57～2.20) | 1.55(0.55～4.34) |
| UMA | 1.31(1.03～1.66) | 1.07(0.80～1.43) |  | 1.46(0.79～2.69) | 1.81(0.83～3.95) |
|  | PCA(n=46) | |  | PCA(n=24) | |
| ≥65 years old | 2.87(1.52～5.40) | 1.16(0.52～2.57) |  | 0.88(0.28～2.73) | 0.90(0.24～3.34) |
| Male | 1.09(0.60～1.98) | 0.63(0.24～1.64) |  | 0.48(0.20～1.17) | 0.72(0.20～2.62) |
| Hypertension | 6.03(2.98～12.19) | 7.54(2.71～20.96) |  | 1.61(0.53～4.91) | 1.69(0.43～6.58) |
| Diabetes | 3.22(1.78～5.80) | 2.80(1.29～6.07) |  | 4.32(1.77～10.55) | 3.59(1.26～10.26) |
| Hyperlipidemia | 1.60(0.86～2.98) | 1.43(0.63～3.21) |  | 1.65(0.63～4.32) | 1.02(0.34～3.09) |
| History of CAD | 2.11(0.64～6.92) | 1.18(0.27～5.29) |  | 1.75(0.55～5.55) | 1.00(0.24～4.24) |
| Smoking status |  |  |  |  |  |
| Never smoker | Reference | Reference |  | Reference | Reference |
| Current smoker | 0.95(0.45～2.00) | 1.31(0.46～3.71) |  | 1.78(0.59～5.38) | 1.75(0.43～7.09) |
| Ex-smoker | 1.02(0.36～2.89) | 0.27(0.03～2.24) |  | 2.04(0.76～5.50) | 0.70(0.14～3.39) |
| BMI (kg/㎡) |  |  |  |  |  |
| Normal weight | Reference | Reference |  | Reference | Reference |
| Underweight |  |  |  |  |  |
| Overweight | 0.89(0.46～1.70) | 0.47(0.20～1.09) |  | 0.96(0.39～2.39) | 0.70(0.24～2.07) |
| Obese | 1.50(0.66～3.40) | 0.56(0.17～1.82) |  | 0.52(0.11～2.43) | 0.51(0.09～2.81) |
| Hyperuricaemia | 1.47(0.71～3.07) | 0.93(0.33～2.61) |  | 0.78(0.25～2.37) | 0.95(0.27～3.35) |
| Hyperhomocysteinemia | 1.05(0.53～2.09) | 0.91(0.41～2.00) |  | 1.22(0.48～3.13) | 1.40(0.48～4.11) |
| Hi-CRP(mg/dl) |  |  |  |  |  |
| 1st tertile(≤0.70 | Reference | Reference |  | Reference | Reference |
| 2nd tertile | 0.76(0.36～1.61) | 0.98(0.37～2.57) |  | 1.10(0.41～3.00) | 1.18(0.33～4.20) |
| 3rd tertile | 1.12(0.57～2.19) | 1.26(0.50～3.18) |  | 1.10(0.39～3.09) | 1.24(0.32～4.80) |
| UMA | 1.81(0.98～3.35) | 1.55(0.72～3.35) |  | 1.42(0.61～3.30) | 1.17(0.42～3.27) |
|  | VA(n=156) | |  | VA(n=55) | |
| ≥65 years old | 3.64(2.52～5.25) | 2.61(1.67～4.05) |  | 1.44(0.57～3.61) | 1.26(0.40～3.99) |
| Male | 1.15(0.83～1.60) | 1.20(0.73～1.98) |  | 0.43(0.23～0.81) | 0.50(0.19～1.32) |
| Hypertension | 3.16(2.25～4.43) | 2.08(1.37～3.17) |  | 0.78(0.41～1.51) | 1.01(0.42～2.45) |
| Diabetes | 2.47(1.77～3.47) | 1.53(1.01～2.34) |  | 1.55(0.85～2.84) | 1.15(0.52～2.55) |
| Hyperlipidemia | 1.74(1.23～2.47) | 1.57(1.03～2.40) |  | 0.61(0.34～1.11) | 0.72(0.32～1.63) |
| History of CAD | 4.45(2.67～7.42) | 1.97(0.95～4.07) |  | 2.19(1.00～4.79) | 1.50(0.51～4.42) |
| Smoking status |  |  |  |  |  |
| Never smoker | Reference | Reference |  | Reference | Reference |
| Current smoker | 0.81(0.52～1.26) | 1.17(0.64～2.14) |  | 2.30(1.09～4.85) | 1.34(0.45～4.00) |
| Ex-smoker | 1.35(0.81～2.25) | 1.13(0.56～2.28) |  | 2.02(0.98～4.14) | 1.15(0.37～3.51) |
| BMI (kg/㎡) |  |  |  |  |  |
| Normal weight | Reference | Reference |  | Reference | Reference |
| Underweight | 0.41(0.06～3.00) | 0.60(0.08～4.55) |  | 1.56(0.29～8.45) | 1.00(0.08～12.17) |
| Overweight | 0.81(0.57～1.14) | 0.61(0.40～0.94) |  | 0.96(0.51～1.81) | 0.80(0.35～1.82) |
| Obese | 0.68(0.38～1.21) | 0.33(0.15～0.71) |  | 0.44(0.15～1.36) | 0.37(0.07～1.89) |
| Hyperuricaemia | 1.81(1.23～2.67) | 1.16(0.71～1.90) |  | 1.08(0.53～2.21) | 1.11(0.45～2.76) |
| Hyperhomocysteinemia | 1.06(0.73～1.53) | 0.95(0.63～1.42) |  | 1.51(0.74～3.08) | 1.18(0.52～2.69) |
| Hi-CRP(mg/dl) |  |  |  |  |  |
| 1st tertile(≤0.70 | Reference | Reference |  | Reference | Reference |
| 2nd tertile | 0.77(0.50～1.20) | 0.92(0.54～1.54) |  | 0.54(0.24～1.20) | 0.64(0.22～1.87) |
| 3rd tertile | 1.52(1.06～2.18) | 1.73(1.08～2.76) |  | 1.35(0.69～2.62) | 1.89(0.70～5.10) |
| UMA | 1.50(1.08～2.10) | 1.10(0.74～1.64) |  | 1.80(0.96～3.36) | 2.86(1.27～6.44) |
|  | BA(n=46) | |  | BA(n=22) | |
| ≥65 years old | 6.99(3.12～15.69) | 3.30(1.29～8.39) |  | 1.76(0.39～7.86) | 0.47(0.06～3.73) |
| Male | 1.60(0.85～3.01) | 1.73(0.68～4.41) |  | 0.45(0.18～1.15) | 0.18(0.03～0.98) |
| Hypertension | 2.60(1.43～4.73) | 2.90(1.30～6.46) |  | 1.45(0.47～4.46) | 3.67(0.49～27.26) |
| Diabetes | 3.22(1.78～5.80) | 2.23(1.08～4.62) |  | 3.12(1.28～7.63) | 3.46(0.90～13.36) |
| Hyperlipidemia | 1.20(0.66～2.19) | 1.32(0.62～2.81) |  | 0.79(0.33～1.94) | 0.28(0.06～1.24) |
| History of CAD | 2.88(1.01～8.20) | 1.39(0.38～5.15) |  | 1.38(0.38～5.01) | 0.86(0.07～10.00) |
| Smoking status |  |  |  |  |  |
| Never smoker | Reference | Reference |  | Reference | Reference |
| Current smoker | 1.13(0.55～2.31) | 1.66(0.56～4.92) |  |  |  |
| Ex-smoker | 1.35(0.52～3.51) | 1.67(0.49～5.72) |  | 2.33(0.90～6.07) | 0.84(0.15～4.69) |
| BMI (kg/㎡) |  |  |  |  |  |
| Normal weight | Reference | Reference |  | Reference | Reference |
| Underweight | 1.12(0.15～8.39) | 1.94(0.23～16.44) |  | 9.34(1.84～47.33) | 138.87(2.45～7,865.21) |
| Overweight | 0.51(0.27～0.98) | 0.31(0.13～0.71) |  | 1.65(0.59～4.61) | 0.78(0.16～3.81) |
| Obese | 0.13(0.02～0.98) | 0.09(0.01～0.68) |  | 1.33(0.33～5.44) | 0.61(0.08～4.67) |
| Hyperuricaemia | 2.14(1.10～4.16) | 1.23(0.52～2.90) |  | 0.39(0.09～1.72) | 0.82(0.13～5.31) |
| Hyperhomocysteinemia | 1.83(0.89～3.77) | 1.57(0.72～3.44) |  | 0.61(0.20～1.83) | 0.38(0.08～1.76) |
| Hi-CRP(mg/dl) |  |  |  |  |  |
| 1st tertile(≤0.70 | Reference | Reference |  | Reference | Reference |
| 2nd tertile | 0.97(0.46～2.05) | 1.65(0.60～4.52) |  | 0.93(0.31～2.80) | 10.95(0.90～133.52) |
| 3rd tertile | 1.48(0.75～2.89) | 2.50(0.98～6.41) |  | 1.41(0.50～3.94) | 13.93(1.14～169.55) |
| UMA | 3.21(1.71～6.05) | 2.65(1.21～5.77) |  | 0.74(0.29～1.85) | 1.20(0.32～4.55) |

*The reference group for the subgroup free of stroke was the same as Supplementary Table 2.

** The reference group for the subgroup with history of stroke was the same as Supplementary Table 5.
